# Extra-pair paternity in the socially monogamous white stork (*Ciconia ciconia*) is fairly common and independent of local density

**DOI:** 10.1038/srep27976

**Published:** 2016-06-22

**Authors:** Sondra Feldman Turjeman, Alejandro Centeno-Cuadros, Ute Eggers, Shay Rotics, Julio Blas, Wolfgang Fiedler, Michael Kaatz, Florian Jeltsch, Martin Wikelski, Ran Nathan

**Affiliations:** 1 Movement Ecology Laboratory, Department of Ecology, Evolution and Behavior, Alexander Silberman Institute of Life Sciences, The Hebrew University of Jerusalem, 91904, Jerusalem, Israel; 2 Department of Molecular Biology and Biochemical Engineering, University Pablo de Olavide, 41013, Seville, Spain; 3 Department of Plant Ecology and Nature Conservation, Institute of Biochemistry and Biology, University of Potsdam, Maulbeerallee 2, 14469 Potsdam, Germany; 4 Estación Biológica de Doñana, Consejo Superior de Investigaciones Científicas (CSIC), C/Américo Vespucio, 41092 Seville, Spain; 5 Deptartment of Migration and Immuno-Ecology, Max-Planck-Institute for Ornithology, D-78315 Radolfzell, Germany 78315; 6 Deptartment of Biology, University of Konstanz, D-78468 Konstanz, Germany; 7 Vogelschutzwarte Storchenhof Loburg e.V., Chausseestr, 18, D-39279 Loburg, Germany; 8 Berlin-Brandenburg, Institute of Advanced Biodiversity Research (BBIB), D-14195 Berlin, Germany; 9 ZALF, Leibniz-Centre for Agricultural Landscape Research, Eberswalder Str. 84, D-15374, Müncheberg, Germany

## Abstract

Although many birds are socially monogamous, most (>75%) studied species are not strictly genetically monogamous, especially under high breeding density. We used molecular tools to reevaluate the reproductive strategy of the socially monogamous white stork (*Ciconia ciconia*) and examined local density effects. DNA samples of nestlings (Germany, Spain) were genotyped and assigned relationships using a two-program maximum likelihood classification. Relationships were successfully classified in 79.2% of German (n = 120) and 84.8% of Spanish (n = 59) nests. For each population respectively, 76.8% (n = 73) and 66.0% (n = 33) of nests contained only full-siblings, 10.5% (n = 10) and 18.0% (n = 9) had half-siblings (at least one nestling with a different parent), 3.2% (n = 3) and 10.0% (n = 5) had unrelated nestlings (at least two nestlings, each with different parents), and 9.5% (n = 9) and 6.0% (n = 3) had “not full-siblings” (could not differentiate between latter two cases). These deviations from strict monogamy place the white stork in the 59^th^ percentile for extra-pair paternity among studied bird species. Although high breeding density generally increases extra-pair paternity, we found no significant association with this species’ mating strategies. Thus although genetic monogamy is indeed prominent in the white stork, extra-pair paternity is fairly common compared to other bird species and cannot be explained by breeding density.

Understanding mating strategies is essential for understanding population structure and dynamics. Life history traits (e.g. age of maturation, migration timing, breeding success), social interactions (e.g. pairing, coloniality), and ecological/environmental factors (e.g. density, climate, land-use), jointly determine mating patterns, which shape individual behaviors and, in the longer term, adaptations^1^,^2^,^3^.

Monogamy was traditionally assumed to be the prevailing breeding strategy of birds^4^, but this generalization has been revised (e.g.^2^,^3^,^5^,^6^,^7^,^8^,^9^) to properly address differences between three types of monogamy: social, sexual and genetic^10^. Social monogamy is a monogamous living arrangement involving a shared territory, nest, or other social trait(s); sexual monogamy, measured by the absence of extra-pair copulations (EPC), is monogamy within sexual interactions; and genetic monogamy requires that all offspring within a given brood share the same genetic parents. While social and sexual monogamy can be measured through behavioral observations; genetic monogamy can only be tested through DNA analysis quantifying parent-offspring and/or sibling relatedness. Deviations from genetic monogamy, often described by the rate of extra-pair paternity (EPP), reflect successful fertilization of an EPC.

Although genetic monogamy could be considered a proof of sexual monogamy, confounding factors such as sperm selection and timing of copulations could still result in findings of genetic monogamy even if a pair is not sexually monogamous^11^,^12^. Conversely, if EPCs occur in hidden or secluded locations, observations alone might lead to a conclusion of sexual monogamy; genetic testing, though, could show a lack of monogamy. Genetic methods allow us to overcome the potential shortcomings of purely observational studies when determining a given species’ mating system^8^,^13^,^14^. Despite the wide-spread occurrence of social monogamy across avian species, recent research has found strict genetic monogamy to be the exception rather than the rule (e.g.^2^,^3^,^5^,^6^,^7^,^8^,^9^,^15^,^16^,^17^,^18^,^19^,^20^).

Because of the potential mismatch between social and genetic monogamy, it is necessary to look beyond observed social patterns and copulations in order to determine a given species’ mating system^13^,^14^. A variety of genetic techniques—including genotyping of single nucleotide polymorphisms, haplotypes, and/or highly polymorphic microsatellites—and comparisons of individual relatedness have become popular in assessing mating systems^21^,^22^,^23^. Parentage analysis of adults and the juveniles in their respective broods is often the easiest method for determining genetic mating systems^24^, but methodological and ecological constraints (e.g. difficulty in trapping adults in natural systems) often preclude procurement of parental genetic matter. In these cases, genetic mating systems can still be elucidated from putative sibling groups (e.g. hatchlings from the same nest) based upon the degree of relatedness between all offspring within a brood^16^,^18^,^19^,^22^,^24^.

A number of ecological factors can influence mating strategies. Species that require bi-parental offspring care are more likely to be socially, sexually, and genetically monogamous because if the male partner doubts his paternity, he might desert the nest or even kill the offspring^8^,^15^,^25^. Contrarily, decreased breeding distance (distance from focal nest to potential extra-pair mate(s)) and increased breeding density (number of breeding pairs per unit area) and breeding synchrony (synchronization in timing of reproductive behavior) can increase the likelihood of EPC and EPP because fertile potential mates are more common and thus more accessible^22^,^26^. Furthermore, due to higher density (in time and space), social mate quality can more easily be compared to that of potential extra-pair mates, leading to EPCs with the intention of improving offspring quality^1^,^8^,^27^,^28^.

The white stork (*Ciconia ciconia*) has traditionally been considered a worldwide symbol of monogamy, modesty, and exemplary joint parenting^29^ that nests and forages either solitarily or in loose colonies or flocks of varying size and density. The species is socially monogamous^30^ and shows high rates of mate fidelity^31^, breeding annually in large nests often reused from year to year^32^. Eggs are laid asynchronously one to four days apart and can be fertilized asynchronously, potentially by more than one male. Clutch sizes range from one to seven with an average of three to four eggs, and both parents incubate and share in brood care^30^,^33^,^34^. Fledging occurs between 58 and 64 days after hatching^30^, and after first breeding at age 2–4 years, adults attempt to reproduce annually^31^. Because the white stork is large, identifiable, and easily observed during breeding, due to a preference to nest in open areas near human settlements, this species is a common subject for scientific studies on reproduction (e.g.^34^,^35^,^36^,^37^,^38^,^39^).

An observational study of white stork mating strategies by Tortosa and Redondo^37^ supported the belief of monogamy. They observed over 4,000 copulations of 43 white stork pairs and found that only 0.45% were EPCs, thus concluding social and sexual monogamy. Genetic monogamy, however, has never been tested in this species.

In this study, we use genetic information to assess the mating strategy of the paradigmatically monogamous white stork. Because of the difficulties associated with trapping adults, genetic data was collected from juveniles and used to analyze within-nest sibling relationships. The use of genetic techniques not only provides insight on the species’ mating biology but also allows for examining the reliability of using behavioral observations (e.g. of copulations) to assess mating strategies. While it is sometimes more difficult to conclusively identify relationships using genetic material obtained only from offspring, the confidence level is comparable to that obtained from analyses including parental material^19^. We hypothesize that, like many other bird species^8^, the white stork is only socially monogamous and likely employs a mixed mating strategy including EPP. We also predict a greater proportion of EPP at higher breeding density as inferred by distance to nearest neighboring nest and by measures of breeding density within the average white stork home range^2^,^27^.

## Results

### Sample collection, DNA extraction, and microsatellite genotyping

DNA samples were collected from white storks nestlings in Germany and Spain. Of the 171 German nests sampled, 22 nests were excluded from analysis because only one nestling was present in the nest at the time of sampling; six nests were excluded because one or more nestlings were too small or weak to be sampled; and 23 nests were excluded because one or more nestlings had fewer than six genotyped loci. Of the 98 Spanish nests that were sampled, 37 were excluded from analysis because only one nestling was present in the nest at the time of sampling and two nests were excluded because one or more nestlings could not be sampled (see Table 1). Genotype frequency tables were constructed from 157 German individuals (this number is lower than the total number of nests sampled because nests in which no sampled nestlings had at least six typed loci (n = 14) could not be included) and 98 Spanish individuals. Relationships analysis was performed on 298 German individuals from 120 nests and on 128 Spanish individuals from 59 nests.

### Tests of Hardy-Weinberg equilibrium and allele frequency database construction

Following initial tests of Hardy-Weinberg equilibrium deviations, two microsatellites (*WS03* and *WS14*) were subsequently removed due to high error rates. P_ID-Sib_ for the microsatellites included in the final database was 0.0000473 for individuals from German sites and 0.00010556 for individuals from Spanish sites, resulting in a value smaller than or in the lower range of the minimum P_ID-Sib_ of 0.001–0.0001 suggested for individual-based genetic studies^38^. The mean numbers of alleles for all loci per region, a measure of polymorphism, were 6.06 and 4.56 in Germany and Spain respectively (see Table 2 for population-wide summaries; see^39^, for locus specific data and rates of genotyping error, null alleles, and allelic dropout).

### Genetic relatedness analysis and classification

**Figure 1:**
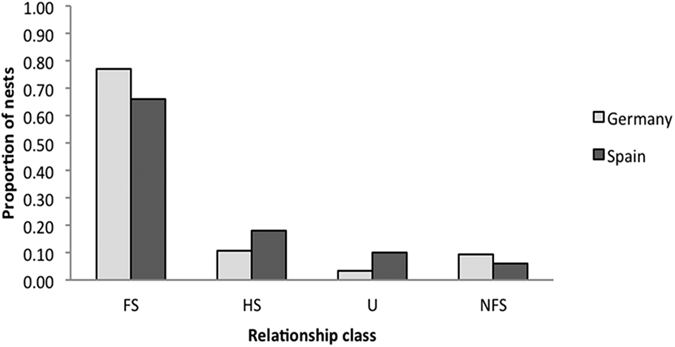
Comparison of nest-wide relationship classes for German and Spanish samples. Proportion of nests per region with only full-siblings (FS), one or more pairs of half-siblings (HS), one or more pairs of unrelated nestlings (U), or one or more pairs of “not full-siblings” (NFS) for cases where full-sibling relationships could be rejected but definitive differentiation between HS and U could not be made. There were no significant differences between populations for any of the relationship classes. N_Germany_ = 95 N_Spain_ = 50.

Overall, for German and Spanish populations, 79.3% (n = 96) and 84.8% (n = 50) of all nests’ nestling relationships were completely classified. In fully resolved nests for Germany and Spain respectively (see Table 3, Fig. 1), 76.8% (n = 73) and 66.0% (n = 33) of nests were comprised only of full-siblings (FS), 10.5% (n = 10) and 18.0% (n = 9) of nests had at least one half-sibling (HS), 3.2% (n = 3) and 10.0% (n = 5) of nests had at least one unrelated individual (U), and 9.5% (n = 9) and 6.0% (n = 3) of nests had at least one “not full-sibling” (NFS; HS and U could not be differentiated).

Two special cases were identified in the German sample when checking the data with field observations (available for only some sample sites in the German sampling). In both cases, a nest was identified to have unrelated individuals due to a prior addition of a foster nestling by local ringers for various conservation reasons (e.g. to save the nestlings when a parent deserted the nest). One such case was removed from the study. The other resulted from poor field records involving a mix-up between ring numbers of native and foster nestlings in a nest. This case was rerun with the relevant individuals and results were included. These cases of genetic results supported by field observations provided external confirmation of the statistical power of our analyses. Based on these findings and associated corrections, analysis and interpretation of German nests refers to a total number of resolved nests as 95 out of 120 sampled.

### Determination of overall breeding strategy

We found no significant difference in the proportion of nests that was successfully classified from the two sampling sites (Fisher exact test, p-value  =  0.4230). The overall proportion of monogamous nests in relation to those with other relationships (106:39) was significantly lower than expected based on the rate of EPC of 0.45% of all copulations, as observed by Tortosa and Redondo^37^ in Spain (Fisher exact test, p-value < 0.0001). The same relationship was found when comparing the German and Spanish samples separately (Fisher exact test, p-value _Germany_ < 0.0001; p-value _Spain_ < 0.0001). These results remain robust when comparing other configurations of the different classes (e.g. FS vs. HS, FS vs. HS+NFS; see Supplementary Table S1).

### Calculation of breeding distances and densities and comparison of EPP rates

In Germany, nearest neighbor distances (*k* = 1) ranged from 0.0092–18.1912 km (mean ± s.d.: 2.33 ± 2.78; median: 1.783; n = 171; *MATLAB*^40^) and in Spain, the range was from 0.00 km (multiple nests in the same tree) to 0.34 km (mean ± s.d.: 0.020 ± 0.047; median: 0.0067; n = 98). Home range density ranged from 0–24 nests in a circle with a radius of 5 km (mean ± s.d.: 7.04 ± 6.73; median: 5) in Germany, and in Spain, home range density for all nests was 81. Division of samples into two clusters resulted in clustering of all German samples (densities 0–24) in the low-density cluster and all Spanish nests (all densities 81) in the high-density cluster.

Multinomial logistic regression testing the effect of breeding density as estimated by minimum breeding distance (to nearest neighboring nest, *k* = 1) on relationship class was not statistically better than the null model considering only the intercept (p-value = 0.48; Cox and Snell Pseudo R^2^ = 0.017). Furthermore, sensitivity tests of distances for *k* = {1, 2, …, 10} nearest neighboring nests did not produce statistically significant models. The chi-square test of breeding density, estimated by home range density, and relationship classification also returned insignificant results (p-value = 0.15); thus, the null relationship of independence cannot be rejected.

## Discussion

Our study shows that the white stork is not a strictly monogamous species, but rather exhibits a mixed mating strategy. While the majority of within-nest relationships are FS, there was a significant deviation from a genetically monogamous reproductive system. Furthermore, despite their reputation as an exemplary model of monogamy, their EPP rate (conservatively estimated to be 13.1%) places white storks in the 59^th^ percentile of EPP for 121 studied bird species^8^.

While it is yet unclear as to why storks have adopted this mixed reproductive strategy, previous studies point to many benefits of EPP that seem relevant based on this species’ behavioral ecology and reproductive biology. Species with long-term social mates, especially migrants which are faced with heavy time constraints on breeding and often make rushed mate selection, can become unsatisfied with their breeding partner^1^. Mate switching to increase genetic quality of subsequent offspring is often risky and can lead to failure to find a new mate or low initial reproductive success. EPC is a less costly alternative than mate switching to obtain “good genes” for offspring as well as to increase genetic diversity among offspring, which is relevant for migratory species that must survive in a wide range of environments (winter, breeding, and stop-over sites)^1^,^8^,^15^.

Although Tortosa and Redondo’s observational approach and the genetic one used in this study are complementary, our findings diverge substantially. This suggests that the original conclusion of negligible incidence of EPC by Tortosa and Redondo^37^ does not accurately predict EPP patterns in the species and leads us to reject the null hypothesis of genetic monogamy. This result is particularly noteworthy for the Spanish population we sampled, which geographically overlaps with the population studied in this previous work^37^. One explanation, specifically relevant to this species for which bi-parental mate care is essential, could be that individuals that seek EPC might do so in a remote location away from the nest, thus reducing the chances of being “caught” and potentially deserted by their social mate. Tortosa and Redondo^37^ only reported copulation events at the nest and thus likely underestimated the actual number of EPCs.

Our findings of EPP and unrelated individuals in the same nests are in line with findings reported in many other avian species (e.g.^6^,^7^,^8^,^9^). The occurrence of nests with unrelated individuals could be a consequence of conspecific brood parasitism, but this is a reproductive strategy that has not previously been reported in white storks. Long-term study of breeding white storks in Germany (*personal observations*) suggest an alternative biological explanation for this surprising finding; in cases where a new or young pair of storks tries to claim a nest early in the season (before the resident pair from the previous year arrives), fights between the new pair and one or both storks of the resident pairs can arise causing the new pair to desert the nest leaving behind one or more eggs not damaged during the fight. This explanation fits with field records from the German sample: of the four U and NFS cases for which there were field records, strong fighting was observed in three nests (75% of the cases). A similar scenario could also explain a proportion of the apparent detected EPP: If mate switching of only one social partner occurs after eggs have been laid by the initial pair and a second clutch is subsequently laid with a new partner, resulting offspring from both clutches will be half-siblings as only one parent has changed. This is the only case in which findings of HS could result in the absence of EPC (though mate switching can often occur as a result of EPC events^37^).

The proportion of EPCs versus total copulations found by Tortosa and Redondo^37^ was 0.45%, but the proportion of individuals in the focal pairs that participated in EPC was much higher. From the article we conservatively estimated that at least 21% of nests showed one or more instance of EPC based on the nine females observed to either mate with males at neighboring nests (6) or to receive extra-pair males at their own nest (3)^37^. This measure is more in-line with our findings. While it is unlikely that such a low proportion of 0.45% extra-pair mating events^37^ would lead to the high proportion of 10.5–24% HS nests we found, in many species, female birds may have control over the success of a copulation in fertilization^27^. Future studies on the breeding system of white storks should consider sperm competition and allocation to determine if and under which ecological circumstances EPCs are more likely to lead to viable offspring than within-pair copulations.

Additionally, we predicted increased EPP with decreased breeding distance and increased local breeding density. Although breeding distances ranged widely in our sample, there were no significant differences in the rate of monogamy at various breeding distances. Furthermore, local density, though very different between regions, had no effect on rates of EPP. These findings suggest that in this species, breeding distance and density does not affect extra-pair paternity, contrary to findings in other avian species^2^,^12^,^18^,^28^. It is important to note, however, that breeding success (as assessed by number of nests with multiple nestlings) was much lower in Spain. This finding could have led to an underestimation of nest-wide EPC and EPP; hence our findings of non-negligible EPP can be conceived as conservative.

In light of these findings and despite previous research asserting that distances to potential extra-pair mates cannot be ignored^28^, we suggest that other mechanisms such as breeding synchrony (resulting from synchronized arrival after long-distance migration) could overshadow the effects of density-dependent reproductive strategies in this species^1^,^41^. Alternative explanations may be related to social mate quality, nest quality, and the characteristics of the individual such as male and female age, experience, body size, and immune system phenotypes (specifically of major-histocompatibility complexes) rather than density effects^1^,^42^,^43^.

Future work examining nest-wide relationships across years for the same populations and in other populations across the white stork breeding range could also facilitate the identification of ecological grounds for a mixed reproductive strategy as EPP can vary both spatially and temporally within species^27^. Furthermore, focusing on non-migratory populations or populations with variations in the length of breeding season, migration flyway, or spring arrival dates to the breeding grounds could provide insight on dominant ecological factors shaping breeding strategies in this species.

## Methods

### Study sites and sample collection

**Figure 2:**
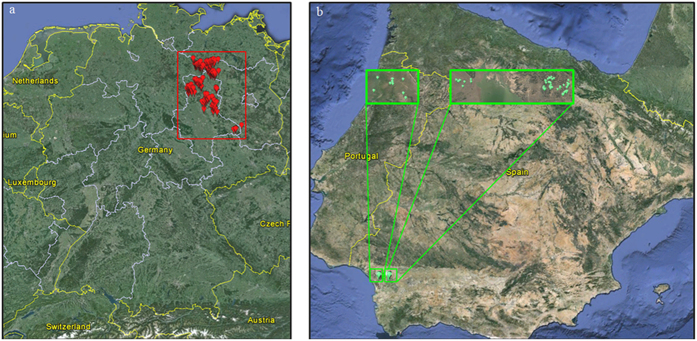
GPS locations of (**a**) German and (**b**) Spanish sampling sites. Maps generated using Google Earth v7.1.5.1557 ((Map data: Google 2016: (**a**) GeoBasis-DE/BKG 2009; Imange Landsat; and Data SIO NOAA USA Navy, NGA, GEBCO and (**b**) Data SIO NOAA USA Navy, NGA, GEBCO; Image Landsat; and US Dept of State Geographer).

DNA samples were collected from white stork nestlings in Germany and Spain during the 2012 breeding season, and sampling locations varied in nest density between and within countries. In northeastern Germany, samples were collected from eight mostly adjacent breeding sites from two federal states (from Saxony-Anhalt: Beuster, Droemling, Gehmen, Kalbe (Milde), Loburg, Magdeburg North, and Magdeburg South and from Brandenburg: Prignitz; see Fig. 2). In southwestern Spain, samples were collected from three nearby breeding sites in the provinces of Huelva and Sevilla (Dehesa de Abajo-Acebuches; Dehesa de Abajo-Encinas; and Matasgordas-Doñana National Park; see Fig. 2). Feathers (five downy chest-feathers per bird) were collected from nestlings during routine regional ringing—something performed every year and not thought to cause excessive stress to nestlings or adult storks (drawing blood is both more risky and time consuming and does not provide substantially higher extraction success rates)^44^. Permits were acquired for all sampling areas and samplings were performed by local experts in accordance with the ethical guidelines as approved by the Autorizacion Expresa from the Consejeria de Agricultura y Pesca (Junta de Andalucía, Spain; reference number 11_24-Blas), the CSIC Bioethics Subcommittee (Madrid, Spain; reference number 11_24-Blas), the Federal State of Brandenburg, Landesamt für Umwelt, Gesundheit und Verbraucherschutz (Brandenburg, Germany; reference number V3-2347-8-2012), and the Federal State of Sachsen-Anhalt: Landesverwaltungsamt Referat Naturschutz, Landschaftspflege (Sachsen-Anhalt, Germany; reference numbers 407.3.3/255.13–2248/2 and 407.3.3/759.12–22482/2).

Feathers were collected no later than three weeks prior to fledging; at this age, nestlings have limited mobility, thus ensuring each nest only contained young that hatched there. Each individual’s feathers were stored separately and grouped by nest. Sampling rates (i.e. proportion of nests visited) in the respective sites were estimated to be 90% in Germany and 30% in Spain and were dependent upon anthropogenic (e.g. avoiding nests situated on high voltage electrical posts) and ecological (e.g. nestlings too small at the time of nest visit to collect samples—there was a wide variation in nestling ages across the breeding region) factors.

### DNA extraction and microsatellite genotyping

DNA was extracted from feathers using a standard NaOH procedure^45^,^46^. Nests with one or more nestlings for which DNA was not successfully extracted from samples (e.g. because collected feathers were too small), for which the polymerase chain reactions (PCR) failed (e.g. due to high concentrations of inhibitors), or in which one or more nestling was not sampled (e.g. too small, weak) were discarded.

Following extraction, each individual was genotyped at 18 polymorphic loci, seven from Shephard *et al*.^47^ (*Cc01, Cc03, Cc06, Cc07, WS03, WS14*, and *WS17;* 2009) and 11 discovered for this project (*Cc10, Cc15, Cc18, Cc37, Cc42, Cc44, Cc50, Cc58, Cc61, Cc69*, and *Cc72*^39^) using PCR (for conditions see^39^,^47^). Appropriate negative controls were used to monitor contamination from foreign DNA, and a random subset of samples was genotyped twice at each locus. Following PCR, genotyping was performed using an ABI PRISM™ 3730xl DNA Analyzer by the *Hebrew University Center for Genomic Technologies*. Allele calling and binning were obtained using *GeneMapper 4.0* software^48^.

### Tests of Hardy-Weinberg equilibrium and allele frequency database construction

After individual genotype construction, an allele frequency database per sample region (i.e. Germany and Spain) was compiled to be used for all analyses related to Hardy-Weinberg equilibrium and as part of the pairwise- and maximum-likelihood relatedness analyses (see next subsection). Databases were comprised of genotypes from one individual per nest for all sampled nests, precluding genetic structure resulting from including related individuals^16^,^18^. The selection of individuals included one bird per nest, choosing the nestling with the most genotyped loci or a random nestling in cases where all individuals had an equal number of typed loci. The separate breeding sites within each country were grouped together as tests of gene flow and structure showed high levels of genetic connectivity (see Table 4 for F_ST_ values between sampling sites).

Tests of Hardy-Weinberg equilibrium (*Cervus 3.0.3*^49^) were performed as were tests of genotyping error (*GIMLET 1.3.3*^50^), null alleles (*FreeNA*^51^), allelic drop-out (*GIMLET*), inbreeding (F_IS_; *Genetix 4.05.2*^52^), linkage disequilibrium (*GENEPOP 4.2*^53^,^54^; with Bonferroni correction for significance), and genetic structure (F_ST_; *Genetix*). The rates of expected and observed heterozygosities, the mean polymorphic information content (PIC) and the probability of identity between unrelated individuals (P_ID_) and related individuals (sibling identity; P_ID-Sib_) were also calculated (*Cervus*).

### Genetic relatedness analysis and classification

Relatedness testing was used to assign pairs of nestlings from the same nest to relationship classes of FS, HS, or U. A category of “NFS” was also defined for cases where FS relationships could be rejected but definitive differentiation between HS and U could not be made.

Relationship class assignment was performed for all nests in which all individuals had at least six genotyped loci, a conservative approach based on Trinca *et al*.^55^, using a two-program congruency approach similar to Miño *et al*.^19^ and de Castro e Souza *et al*.^16^. German and Spanish samples were analyzed separately. First the program *ML-Relate*^56^ was used to determine the most likely relationship between nestling pairs using a maximum likelihood approach. Then the hypothesis testing tool, based on simulations, was used to determine statistically significant relationships between pairs in a manner similar to that used in Miño *et al*.^19^ (see Supplementary Fig. S1 for exact detailing of the procedure used).

The second program used for this congruency approach was *Colony2*^57^ (*v. 2.0.5.4*) with which full families were also reconstructed. For all runs, the parameters included were: female polygamy, male monogamy (while both parents could be polygamous, because population-wide relatedness was not examined and thus a single individual’s paternity in multiple nests is irrelevant, male monogamy was chosen for computational simplicity and to increase sensitivity), strong size prior for family size based on an average brood size of 2.41 (Germany) or 2.17 (Spain), error rates based on GIMLET results, and a ‘very long’ run using the ‘full likelihood method’. Three runs were performed using different random number seeds. ‘Maternal exclusions’ were provided based on the nests from which the feathers were collected such that the program only checked within nest relationships and not population-wide relatedness. The program produced sibship clusters with putative parent identifications for each nestling and from this information, nestling relationships were extracted. These relationships were compared with those resulting from *ML-Relate*.

**Figure 3:**
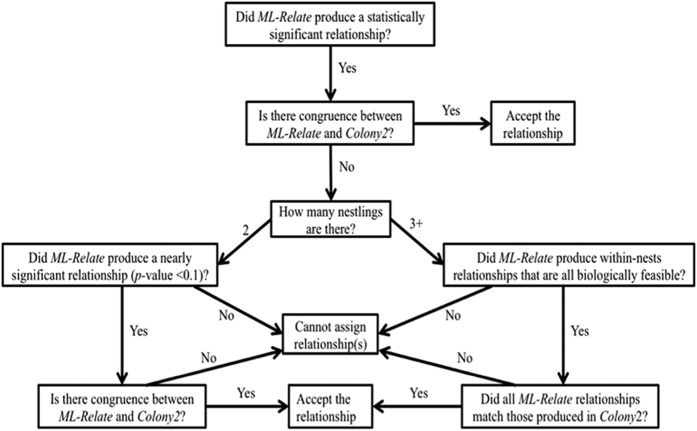
Decision tree for assigning relationships based on the *ML-Relate* | *Colony2* two-program congruency method. In cases where *Colony2* produced the same relationships as those found significant in *ML-Relate*, the suggested relationship was accepted. In cases where *ML-Relate* could not produce statistically significant relationships, if the most likely relationship was nearly significant (in cases of two nestlings per nest) or was biologically correct based on other significant relationships within the nest (e.g. in a nest of three nestlings, when all three pair-wise comparisons were classified as full-sibling, but only two pair-wise comparisons were statistically significant), and this set of relationships matched the family-wise relationship produced in *Colony*2, the relationships were accepted. In cases where no reasonable conclusion could be produced based on *ML-Relate* no classification for the pair was made.

In cases where *Colony2* produced the same relationships as those found significant in *ML-Relate* (87.7%; n = 128), the suggested relationship was accepted. In cases where there was no congruence between programs (n = 18), decisions were reached based upon the decision tree in Fig. 3. Nests were classified FS if all sibling pairs had FS relationships and were classified HS, U, or NFS if one or more sibling pairs had a HS, U, or NFS relationship, respectively.

### Determination of overall breeding strategy

Fisher’s exact test of FS versus all other relationship classes combined was used to determine if white storks deviate significantly from genetic monogamy. Fisher’s exact test was also used to assess differences in assignment success between German and Spanish samples (*SPSS v. 21.0*^58^).

### Calculation of breeding distances and densities and comparison of EPP rates

Breeding density was estimated using two techniques: (1) Average distances from a focal nest to the *k*  = {1, 2, …, 10} nearest neighboring nests were used to approximate breeding distances to potential mates for each nest. (2) Local density was calculated for each nest by counting all of the nests within a five km radius of the focal nest (nesting home range of the white stork^59^,^60^; *MATLAB 2013b*^40^). Two German nests were removed from this analysis because their locations were not recorded at the time of sampling. Additionally, because the sample taken from Matasgordas represents only a very small proportion of the stork nests at the Doñana National Park in Spain, we excluded these nine nests from this particular analysis to avoid a strong deviation from the actual regional density.

Multinomial logistic regression was used to determine the effect of breeding distance to the nearest neighboring nest on relationship classes. Sensitivity testing using the mean distance of *k*  = {1, 2, …, 10} nearest neighbors was employed (*SPSS*). To determine the effect of breeding density on relationship classes, a chi-square test for independence was used as breeding density values were not normally distributed. Nests were categorized as either low- or high-density based on the *kmeans* clustering function in *MATLAB*, and all four relationship classes were included (*SPSS*).

## Additional Information

**Accession codes**: Cc01: *GenBank FJ440852*; Cc03: *GenBank FJ440854*; Cc06: *GenBank FJ440857*; Cc07: *GenBank FJ440858*; Cc10, Cc15, Cc18, Cc37, Cc42, Cc44, Cc50, Cc58, Cc61, Cc69, and Cc72: *GenBank KT232056-KT232066*; WS17: *GenBank AY268097*.

**How to cite this article**: Turjeman, S. F. *et al*. Extra-pair paternity in the socially monogamous white stork (*Ciconia ciconia*) is fairly common and independent of local density. *Sci. Rep.* **6**, 27976; doi: 10.1038/srep27976 (2016).

## Supplementary Material

Supplementary Information

## Figures and Tables

**Table 1:** Summary of sample sizes per population.

Population	Total nests sampled	Nests excluded because nest had only one nestling	Nests exclude because one or more nestling was not sampled	Nests excluded due to low genotyping success of one or more nestlings	Number of nests included in relationship analysis	Number of genotyped nestlings in analysis
Germany	171	22	6	23	120	298
Spain	98	37	2	–	59	128

Quantification of samples per study site and nests included in the analyses, including support for exclusion of samples from the final dataset.

**Table 2:** Summary of microsatellite diversity and sensitivity.

Sample Region	Mean no. of alleles	Loci with significant deviations from HWE	H_Obs_	H_Exp_	PIC	P_ID_	P_ID-SIB_	Inbreeding (F_IS_†)	Number of individuals typed	Mean proportion typed per locus
Germany	6.06	Cc50*; Cc58***	0.5101	0.5393	0.4888	3.16E-11	4.73E-05	0.04941	57	0.9601
Spain	4.56	–	0.4916	0.5019	0.4485	4.77E-10	1.06E-04	0.02137	98	0.9821

Tests for microsatellite diversity and sensitivity including the mean number of alleles per locus sampled, deviations from Hardy-Weinberg equilibrium (HWE), mean observed and expected heterozygosities (H_Obs_ and H_Exp_, respectively), polymorphism information content (PIC), probabilities of identity (P_ID_) and sibling identity (P_ID-Sib_), inbreeding rates (F_IS_), and the number of individuals included in the population-specific databases (which include one individual per nest sampled, so long as genotyping success rate was not less than 10 loci). Significance is denoted as follows: *p-value = 0.01 to 0.05; **p-value = 0.001 to 0.01; ***p-value < 0.001, unmarked: not significant.

^†^1,000 permutations used for F_IS_ tests of significance.

**Table 3:** Relatedness classifications for nests from German and Spanish samples.

	FS	HS	U	Not FS	n_resolved_	n _total_
Germany	73 (76.8%)	10 (10.5%)	3 (3.2%)	9 (9.5%)	95 (79.2%)	120
Spain	33 (66.0%)	9 (18.0%)	5 (10.0%)	3 (6.0%)	50 (84.7%)	59
Total	106 (73.1%)	19 (13.1%)	8 (5.5%)	12 (8.3%)	145 (81.0%)	179

Nest-wide classifications were of: only full-siblings (FS), one or more pairs of half-siblings (HS), one or more pairs of unrelated nestlings (U), or one or more pairs of “not full-siblings” (NFS) for cases where full-sibling relationships could be rejected but definitive differentiation between HS and U could not be made. n _resolved_ refers to the number of nests for which a class could be assigned and n _total_ represents the total number of nests assessed. Number in () is the percentage of category based on n _resolved_. For n _resolved_, () is the percentage resolved based on n_total_.

**Table 4:** Summary of genetic structure (F_ST_) between (a) German and (b) Spanish sampling sites.

	Droemling	Gehmen	Kalbe (Milde)	Loburg	Prignitz	Magdeburg North	Magdeburg South
(a)							
Beuster	0.01985*	0.01285	−0.00655	0.00356	0.00710*	−0.02634	0.03766***
Droemling		0.00636	0.01553	0.01742	−0.00113	−0.01099	0.03130*
Gehmen			0.02449	0.02786*	0.01461	−0.00606	0.03266
Kalbe (Milde)				0.01767	0.00720	−0.02501	0.03157*
Loburg					0.00188	−0.02065	0.01402
Prignitz						−0.01641	0.01078
Magdeburg North							−0.00843
(b)							
	**Dehesa de Abajo, Encinas**	**Matasgordas National Park**					
Dehesa de Abajo, Acebuches	0.00046	0.00434					
Dehesa de Abajo, Encinas		0.00687					

F_ST_ values for comparisons between sampling sites in (a) Germany and (b) Spain. Significance was determined using permutations (1000) and is denoted as follows: *p-value = 0.01 to 0.05; **p-value = 0.001 to 0.01; ***p-value < 0.001; unmarked: not significant.
