# Supplementary material for: Extra-pair paternity in the socially monogamous white stork (Ciconia ciconia) is fairly common and independent of local density
Source: Sci Rep. 2016 Jun 22;6:27976. doi: 10.1038/srep27976 (PMC4916429; doi:10.1038/srep27976)
Supplement: Supplementary Information [file srep27976-s1.pdf]

Extra-pair paternity in the socially monogamous white stork (*Ciconia ciconia*) is fairly common and independent of local density

Sondra Feldman Turjeman[1*], Alejandro Centeno-Cuadros[1,2], Ute Eggers[3], Shay Rotics[1], Julio Blas[4], Wolfgang Fiedler[5,6], Michael Kaatz[7], Florian Jeltsch[3,8,9], Martin Wikelski[5,6], and Ran Nathan[1]

Supplementary Information

Table S1.

There is strong support against the claim of conspecific brood parasitism in cases of "unrelateds" (U), thereby suggesting that occurrence of U is not an active reproductive strategy but rather a result of other species-relevant behaviors. Because of the cases of "not full siblings" (NFS), though, comparing only full-siblings (FS) and half-siblings (HS) leads to difficulty in interpretation. If only U is removed from the Fisher exact tests, the actual proportion of HS could be overestimated by including NFS; similarly, if both U and NFS are removed, there is a risk of underestimating the true proportion of HS. When these two versions of the test are performed, the results of the tests are in line with those when U and NFS are included. All combinations of Fisher exact tests are presented below.

	Relationship Class 1	$N_{Germany}$	N_{Spain}	Relationship Class 2	$N_{Germany}$	N_{Spain}	*p*-value (2-tailed)
1	Resolved nests	95	50	Unresolved nests	25	9	0.4230
2	FS	73	33	All other classes	22	17	0.1730
3	FS	73	33	HS	10	9	0.1924
4	FS	73	33	U	3	5	0.1145
5	FS	73	33	NFS	9	3	0.7539
6	FS	73	33	HS+NFS	19	12	0.5150
7	FS	73	33	U+NFS	12	8	0.4454
8	HS	10	9	U	3	5	0.6776
9	HS	10	3	NFS	9	3	0.3201
10	HS	10	3	U+NFS	12	8	0.4559
11	U	3	5	NFS	9	3	0.1675
12	NFS	9	3	Unresolved nests	25	9	1.0000

Figure S1.

Testing was performed such that in cases where the most likely relationship produced by *ML-Relate* was that of full-sibling (FS), half-sibling (HS) was used as an alternative

hypothesis to determine that the most likely relationship of FS was in fact statistically significant (no conclusion (N.C.) was assigned for *p*-value > 0.05). In cases when HS was the most likely relationship, it was used as the null hypothesis (Miño et al. 2011; de Castro e Souza et al. 2013) and tested against the alternative hypothesis of FS (no conclusion (N.C.) was assigned for *p*-value > 0.05). In cases where a parent offspring (PO) or an unrelated (U) relationship was determined as a most likely relationship, the *a priori* relationship was that of FS based on Tortosa and Redondo's observational study (1992) and the alternate hypothesis was that of HS. If HS could not be rejected, PO or U, respectively, was used as the *a prioi* relationship against FS and HS to see if one of the alternative relationships could be rejected *(ML-Relate* works on the basis of rejecting alternative hypotheses: “Interpretation: If the *p*-value is small, you can conclude that the putative relationship fits the data significantly better than the alternative relationship”; Kalinowski et al. 2006). In cases where PO, an impossible finding based on data collection, was the most likely relationship, if either FS or HS could be rejected, the other (non-rejected) relationship was accepted as the correct relationship (no conclusion (N.C.) was assigned for *p*-value > 0.05 or when both FS and HS were rejected against PO). In cases where U was the most likely relationship, if both alternative relationships were rejected, U was concluded as the final relationship. If only FS could be rejected, a relationship of not full-siblings (NFS) was assigned. Otherwise no relationship was defined (N.C.).

Figure S1.

Step 1: Hypothesis Testing

**Most likely Relationship**	FS		HS		PO or U	
**H_0**	FS		HS		FS (based on Tortosa and Redondo 1992)	
**H_1**	HS		FS		HS	
	p<0.05	p>0.05	p<0.05	p>0.05	p<0.05	p>0.05
***ML-Relate* Relationship**	FS	N.C.	HS	N.C.	FS	See "Step 2"

Step 2: Further PO & U Hypothesis Testing

**Most likely Relationship**	PO				U			
**Hypothesis Test**	Test 1		Test 2		Test 1		Test 2	
**H_0**	PO		PO		U		U	
**H_1**	FS		HS		FS		HS	
**Test 1 *p-value***	p>0.05	p>0.05	p<0.05	p<0.05	p>0.05	p>0.05	p<0.05	p<0.05
**Test 2 *p-value***	p>0.05	p<0.05	p<0.05	p>0.05	p>0.05	p<0.05	p<0.05	p>0.05
***ML-Relate* Relationship**	N.C. (neither FS or HS rejected)	FS (HS rejected)	N.C. (both FS and HS rejected)	HS (FS rejected)	N.C. (neither FS or HS rejected)	- (will not occur)	U (both FS and HS rejected)	NFS (FS rejected)
